# A Dynamic Foraging Habitat Distribution Estimate for Green Turtles in the Great Barrier Reef

**DOI:** 10.1002/ece3.73146

**Published:** 2026-02-25

**Authors:** Emily Webster, Stephanie Duce, Colin Limpus, Nicholas Murray, Toby Patterson, Richard Pillans, Takahiro Shimada, Mark Hamann

**Affiliations:** ^1^ James Cook University College of Science and Engineering Douglas Queensland Australia; ^2^ Aquatic Threatened Species Program, Queensland Department of Environment, Tourism, Science and Innovation Brisbane Queensland Australia; ^3^ CSIRO Environment Hobart Tasmania Australia; ^4^ CSIRO Environment St. Lucia Queensland Australia

## Abstract

A detailed understanding of how protected species use their habitats can guide management interventions in areas of high human use. For marine turtles, different food availability and physical habitat characteristics can underpin turtle presence at anthropogenically modified compared to unmodified sites. We develop telemetry‐based habitat models with boosted regression trees to identify the environmental characteristics underpinning foraging habitat suitability for green turtles in the Great Barrier Reef region. We fit models to green turtle Fastloc GPS tracks from both modified and unmodified inshore foraging sites and using pseudo‐absences (simulated correlated random walks). We assess model performance by the ability to predict known foraging areas, true skill statistic, explanatory power (percent deviance explained) and predictive skill (AUC) of the models. We then predict potentially suitable foraging areas for green turtles in the Great Barrier Reef region using the model for unmodified habitats. Our model highlights shallow nearshore environments and midshelf reefs as important foraging areas for green turtles. These areas are likely affected by dynamic floods, development, and turbidity. In 2022, 46.6% of predicted suitable habitat fell within habitat protection zones, and 16.5% in Marine National Park Zones of the Great Barrier Reef Marine Park. A detailed foraging distribution of the species has not previously been compiled at this regional scale. Identifying biophysical drivers of habitat suitability can inform identification of possible foraging habitat in less data rich regions in Australia and overseas. Evaluating changes over time in habitat distribution provides insights into the degree to which broad‐scale environmental changes and anthropogenic activities influence the condition and function of habitats, even within protected area boundaries.

## Graphical abstract

We develop telemetry‐based habitat models with boosted regression trees to identify the environmental characteristics underpinning foraging habitat suitability for green turtles in the Great Barrier Reef region. We then predict potentially suitable foraging areas for green turtles in the Great Barrier Reef region. A detailed foraging distribution of the species has not previously been compiled at this regional scale.

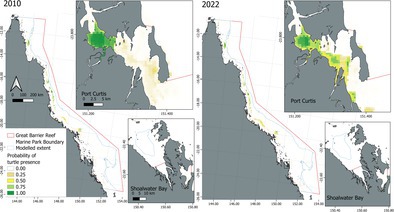

## 1 Introduction

Detailed spatio‐temporal information about how animals use their habitats advances ecological knowledge and can be incorporated into systematic conservation planning to manage harmful human‐wildlife interactions (Hays et al. 2019) and human activities that mediate habitat quality. For protected species, mitigating human‐wildlife interactions in areas outside of marine park jurisdiction, such as within designated port areas, is a high priority (Queensland Department of Environment and Science 2021). Human impacts on habitats can affect populations of species at multiple life stages.

Habitat quality underpins population health for the green turtle *Chelonia mydas* (Limpus and Nicholls 2000, 1988) and is mediated by natural and anthropogenic disturbances. Foraging habitat for green turtles includes subtidal and intertidal seagrass or algal flats, coral or rocky reefs and estuarine areas. Green turtles occupy these neritic foraging areas across age and sex cohorts and display high foraging site fidelity (Shimada et al. 2019).

The majority (97%) of eastern Queensland coastal waters lie within state or federal protected areas (Dryden et al. 2008; Limpus et al. 2013). Nevertheless, in eastern Queensland foraging areas, threats to green turtles include boat strike, entanglement and ingestion of marine debris, legal and illegal harvest and habitat degradation resulting from run‐off, frequency and severity of floods and physical modification to habitat from coastal and marine developments (Limpus et al. 2013; Kennett et al. 2004; Limpus et al. 2005; Limpus 2008), with the impacts of coastal degradation the most problematic to quantify and address.

There is evidence to suggest that green turtles are not displaced by chronic and acute disturbances, with turtles remaining in place during hurricanes and in degraded foraging sites (Lamont et al. 2021; Matley et al. 2019; Flint et al. 2015). However, satellite tracking studies in Queensland have shown that rates of emigration in green turtles were higher within a developed port compared to relatively pristine habitats (Pillans et al. 2021, 2022). Also, in eastern Australia, following flood years, the influx of chemicals and sediment into coastal foraging grounds generally leads to reductions in the quantity/quality of turtles' benthic food resources, most famously, seagrass (Limpus and Nicholls 2000). This has been noted to cause a decline in female turtle breeding rates (Limpus and Nicholls 2000). Therefore, knowing where foraging habitats are distributed is important to appropriately manage new and existing human activities in, and adjacent to, these habitats to minimise risks to health, reproduction and survivorship of turtles and maintain the health of their habitats.

Obtaining sufficiently detailed species location data to determine the distribution of habitats can be challenging. Delineating foraging areas is an important component of stock assessments and the definition of Key Biodiversity Areas (IUCN 2016) and Biologically Important Areas (DCCEEW 2023), however, the location of these habitats is largely unknown in areas that are difficult to access or far away from human population centres. Aerial survey of the Great Barrier Reef region (Cleguer et al. 2022) captures a snapshot of adult marine turtle distribution, but does not distinguish between foraging, migrating and breeding life stages, and therefore cannot be used to make inferences about habitat use and resource requirements.

Satellite telemetry can provide observations of highly mobile species with high spatial and temporal accuracy, but it is expensive to collect, meaning sample sizes are generally small and accuracy can vary depending on the technology used, satellite coverage and signal strength. Habitat information may be derived from remotely sensed data or modelled, but collating this data collected for different purposes using different methods can make it challenging to match the scale, resolution and extent of datasets. Similarly, not all habitat variables are measurable and not all environmental data sources are publicly available. Nevertheless, integrating information from multiple biophysical and environmental datasets can improve estimates of spatio‐temporal patterns of species distribution and enable habitat boundaries to be developed (Liang et al. 2023).

This study targets actions for the management of green turtles that were explicitly identified by the Queensland Marine Turtle Conservation Strategy in 2021 (Queensland Department of Environment and Science 2021): ‘identify important inshore, shallow foraging grounds’ (p36 and p41, for the sGBR and nGBR genetic stocks respectively); ‘assess the residual risk to those areas that are not currently protected and consider the most appropriate habitat mechanisms to ensure protection’ (p36 and p41 for sGBR and nGBR), and ‘recording changes over time … in foraging habitat’ (p24).

Based on these identified needs, this study aims to integrate green turtle satellite telemetry with publicly available spatial and spatio‐temporal environmental datasets to develop telemetry‐based habitat models that establish:

1. What characteristics make foraging habitat suitable for green turtles in anthropogenically modified and unmodified habitats?
2. What is the distribution of suitable unmodified foraging habitat for green turtles in the Great Barrier Reef inshore region?
3. How has the extent and distribution of suitable unmodified foraging habitat in the region changed over time?
4. What proportion of these sites is protected?

To our knowledge, previous attempts to ascertain the distribution of green turtle foraging habitat at a regional scale have relied on expert input rather than a quantitative approach (Dobbs et al. 2007). Opportunities for threat mitigation arise where habitat distributions overlap with expanding anthropogenic footprints. Therefore, this study serves as a baseline for assessing the risk of human‐wildlife interactions based on modelled distribution of green turtle foraging habitat in space/time.

## 2 Methods

### 2.1 Study Sites

**FIGURE 1:**
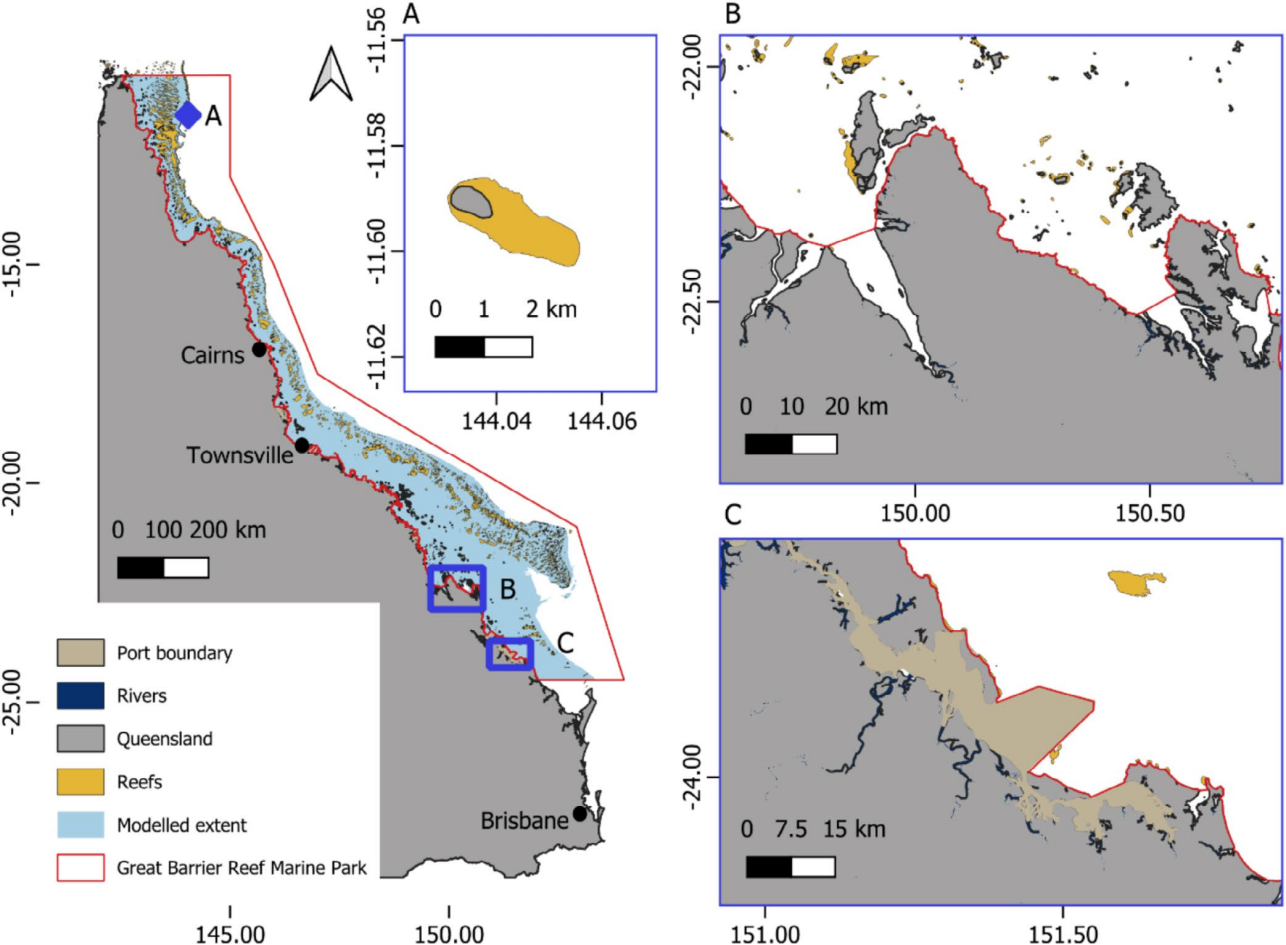
Map of Queensland with blue overlays indicating three study sites where green turtles were tagged, (A) Raine Island, (B) Shoalwater Bay and (C) Port Curtis.

Turtles were tracked during foraging at Port Curtis, Shoalwater Bay and post‐nesting from Raine Island (Figure 1). Port Curtis (Figure 1C) is a major multi‐commodity port in central Queensland, containing extensive seagrass meadows and receiving freshwater inputs from the Fitzroy and Calliope Rivers. Port Curtis is described in detail in (Webster et al. 2022). Shoalwater Bay (Figure 1B) in central Queensland is a heritage‐listed, shallow estuarine area receiving numerous freshwater inputs. As a remote military training area, Shoalwater Bay experiences minimal disturbance from human activities, except during training exercises. The bay is bordered by mangroves, contains fringing reefs, rocky shores and supports extensive seagrass meadows.

Raine Island (Figure 1A), 120 km from the mainland in the northern Great Barrier Reef, is the largest green turtle rookery in the world. Green turtles nesting at Raine Island largely pertain to the northern Great Barrier Reef genetic stock and migrate to Raine Island from foraging grounds along the shallow coast of eastern Australia or coral reefs. For this study, we considered Port Curtis to be highly anthropogenically modified and Shoalwater Bay and Raine Island to be unmodified sites.

### 2.2 Satellite Tracker Deployment

Green turtles were captured as detailed in (Webster et al. 2022; Gredzens et al. 2014), using the rodeo method described by Limpus and Reed (1985) or using blocking nets set on the falling tide. Turtles were tracked with Fastloc GPS, with a mean error 40 m (Dujon et al. 2014) and/or ARGOS tags, with mean error ranging < 1 km to > 10 km, opportunistically between 2010 and 2021, resulting in a mixed sample of sex and maturity cohorts across the three study sites (Appendix S1, Figure 1). Sex, maturity and curved carapace length (CCL) were recorded for each tracked individual. Turtles were sexed via laparoscopy, or sex was determined to be ‘unknown’. Capture of turtles, transmitter attachment and data collection for this study were approved by either the JCU Animal Ethics Committee or the Department of Agriculture and Fisheries Ethics Committee and conducted within an approved Queensland Government project. The tracking dataset included 85 turtles at Port Curtis (mean ± SD CCL = 84.78 ± 21.66 cm), four at Shoalwater Bay (mean ± SD CCL = 92.93 ± 2.33 cm) and five who migrated to foraging grounds from Raine Island (mean ± SD CCL = 102.89 ± 2.72 cm, Appendix S2). The Port Curtis turtles comprised 35 females, 36 males and 14 unidentified sex. Eighteen were juveniles, 24 were subadult turtles and the remainder were mature (49% of turtles in modified habitats were immature). At Shoalwater Bay one was female and three were males, only one of the four was a juvenile. The Raine Island turtles were all post‐nesting adult females (11% of turtles in unmodified habitats were immature).

### 2.3 Tracking Data Filtering

We discarded the first 24 h of the tracks from Port Curtis and Shoalwater Bay. We removed duplicates and empty rows, spurious locations determined from unrealistic travel speeds and turning angles (max speed 9.9 km/h and threshold inner angle 90 degrees), and locations falling above the high tide line using the SDLfilter R package (Shimada et al. 2012). We treated tracks as independent when a single track contained gaps of more than 72 h between successive locations, and only retained independent tracks consisting of more than 20 locations. We used both Fast‐loc GPS and ARGOS locations where available, but only retained ARGOS points with location classes 1, 2 and 3. We removed nesting and post‐nesting tracks of Raine Island turtles by visually identifying the end of directed travel from Raine Island. We also removed possible breeding migrations of Port Curtis turtles identified as a long‐distance (greater than 150 km) departure from their release site to known breeding sites for the species during summer months. The filtered tracking data contained 21,578 ARGOS and 56,409 Fast‐loc GPS locations.

### 2.4 Telemetry‐Based Habitat Models

To identify the environmental characteristics that make foraging habitat suitable for green turtles we developed telemetry‐based habitat models using the tracking data. These models combine species presences from telemetry data and pseudo‐absences generated by models detailed below with environmental data to first identify environmental predictors underpinning species presence and then predict distributions in space and time. We adopted the approach of Hazen et al. 2021 (Hazen et al. 2021) whereby telemetry‐based habitat models are constructed using interpolated locations of tracked animals as presences, and pseudo‐absences are generated using correlated random walks. Though similar to species distribution modelling, telemetry‐based habitat modelling does not use abundance or true presence data to simulate a realised distribution of a species. Instead, presences derived from tracking are spatially and temporally autocorrelated and therefore spatially biassed and pseudo‐absences are simulated to control for these biases. For example, pseudo‐absences can be generated from simulations that mimic the movement process so that both presences and pseudo‐absences have the same autocorrelation structure. The model output reflects the habitat conditions that best explain turtle presence.

#### 2.4.1 Presences and Pseudo‐Absences

We derived presences from the tracking data by interpolating locations at regular intervals, accounting for telemetry error, with time‐varying move persistence models in the aniMotum R package (Jonsen et al. 2023). We have attempted to reduce autocorrelation and imperfect detection biases with state space models (movement persistence models), which interpolated the irregularly sampled data points to produce an estimated location at least 12‐h intervals (Jonsen et al. 2023; Braun et al. 2023). We assumed the data had symmetric spatial error, using aniMotum's default error margins for ARGOS locations, and estimated error from (Webster et al. 2022) for Fast‐loc GPS. For each independent track, we examined state‐space models with diagnostic visualisations of (1) a time‐series, (b) qq‐plots and (c) autocorrelation functions. By visual examination of candidate state‐space model outputs we determined the most appropriate timestep, 12 h where possible or lower resolution models for tracks with sparser data (24 or 48 h). Sparse data resulted in non‐convergence or unrealistic patterns (straight lines or perfect loops), which were discarded.

We generated pseudo‐absences via correlated random walks (CRW, i.e., where the animal could have gone based on track simulations defined by step lengths and turning angles from the interpolated tracking data; each simulated point is overlayed with the ‘worldHires’ map from the mapdata R package (Becker et al. 2022) and if the point is on land it is not used and a new point is generated, Appendix S2) as per (Hazen et al. 2021). CRW models were used to infer drivers of turtle presences on local scales. Our approach assumes presences and pseudo‐absences are independent, despite tracking data being inherently autocorrelated in space and time. Turtles tracked for longer are therefore more represented in the presence/pseudo‐absences, as are turtles tracked in Port Curtis compared to other sites (Appendix S2).

#### 2.4.2 Environmental Predictors

To investigate the relationship between probability of turtle presence and environmental conditions, we developed a set of environmental predictor layers from which to ascertain the conditions at each tracked location in space and time. We catalogued freely available environmental datasets with spatial coverage of the coastal Great Barrier Reef region, and selected variables with known relevance to green turtle foraging ecology based on current literature, with at least 4 km spatial resolution, and appropriate temporal coverage of the tracking period (static datasets developed since 2010 or time‐varying spatial data covering late 2010 to late 2019). Selected datasets are summarised in Table 1.

**TABLE 1:** Selected datasets from which environmental predictors were derived for telemetry‐based habitat modelling.

Data source	Variables selected	Resolution	Temporal resolution	Temporal range	Scale	Append method or transformations applied	Rationale for inclusion
eReefs hydrodynamic model (Herzfeld et al. 2016; CSIRO 2023a)	Temperature, Salinity, Mean seawater velocity, Mean wind speed	4 km	Daily	September 2010 to present	GBR	Extracted values at corresponding pixels (in space and time) to presences/pseudo absences	Green turtles are ectotherms but may occupy a limited preferred range for thermoregulation (Madrak et al. 2016; Crear et al. 2016). Green turtles have tolerance of a range of temperatures (Musick and Limpus 1997). Juveniles have fidelity to feeding sites where temperature is stable (Musick and Limpus 1997; Chambault et al. 2016). There is a small temperature range at Port Curtis (Shimada et al. 2019, 2016). Chronically high temperatures would affect primary producers (Rasheed et al. 2014; Campbell and McKenzie 2004). Green turtle tolerance of a range of salinity values (Limpus 2008). Tides provide intermittent access to intertidal habitat (Pillans et al. 2021). Currents aid passive transport (Senko et al. 2010). In Port Curtis currents are primarily tidal. Possible windborne navigational cues (Akesson et al. 2003; Luschi et al. 1999). Wind direction affects water movement—currents and wave action.
eReefs biogeochemical model (CSIRO 2023b; Baird et al. 2020)	Secchi depth, Ecology fine inorganics (EFI i.e., sum of fine sediment and mud concentrations, derived as total suspended solids/1000), Large zooplankton nitrogen	4 km	Daily	December 2010 to April 2019	GBR	Extracted values at corresponding pixels (in space and time)	Chronically high values affect primary producers (Rasheed et al. 2014; Campbell and McKenzie 2004). Sediment and chemical influx and resuspension affect primary producers and are associated with toxicity and pathogens in turtles (Flint et al. 2010). Zooplankton as a food source (Esteban et al. 2020; Arthur et al. 2007).
Digital Earth Australia (Lymburner et al. 2020)	Mangrove canopy cover (Landsat): ‘Distance to mangroves’	30 m	Annual	1987–2022	National	Distance to pixels of at least 20% canopy cover in corresponding year	Mangroves as a food source (Limpus and Limpus 2000).
Geoscience Australia Intertidal Model Relative Extents (Geoscience Australia 2016)	Tidal exposure	25 m	Static	1987–2015	National	Extracted values at corresponding pixels (in space only)	Intermittent access to intertidal habitat (Senko et al. 2010).
Carter (NESP TWQ Project 5.4, TropWATER, JCU) (Carter et al. 2021)	Seagrass probability	30 m	Static	1984–2023	Coastal GBR	Extracted values at corresponding pixels (in space only)	Seagrasses as a food source (Bjorndal 1997, 1980).
Carter (NESP TWQ Project 5.4, TropWATER, JCU) (Carter et al. 2021)	Seagrass community type	Categorical	Static	1984–2023	Coastal GBR	Overlap with polygon feature	Food source (Bjorndal 1997, 1980)
Heap and Harris (2008)	Seafloor geomorphological feature types	Categorical	Static	2008	National	Overlap with polygon feature	Structural features for foraging, resting and predator avoidance (Chambault et al. 2020; Limpus and Fitzsimmons 2020).
Queensland transport and main roads (Department of Transport and Main Roads 2022)	Recreational boating facilities: ‘Distance to boat ramps’	Categorical	Static	2021	Queensland	Distance to pixels containing features	Modified structural features for foraging, resting and predator avoidance (Chambault et al. 2020; Limpus and Fitzsimmons 2020).
Geoscience Australia Intertidal Model Relative Extents (Geoscience Australia 2016)	Distance to coast	25 m	Static		Queensland	Distance to nearest pixel exposed at highest 80–100% of the observed tidal range (land)	Documented as influential in prior studies.
GBRMPA GBR features (Great Barrier Reef Marine Park Authority 2017)	Distance to reefs	Categorical	Static	2003	Queensland	Distance to nearest ‘reef’ pixel	Food source (Forbes 1996, 1994). Structural features for foraging, resting and predator avoidance (Chambault et al. 2020).
Geoscience Australia Surface Hydrology Polygons (Crossman and Li 2015)	Distance to rivers	Categorical	Static	2015	Queensland	Distance to nearest ‘river’ pixel	Diseases in coastal populations are associated with chemical and sediment runoff from river outflows (Flint et al. 2015, 2010; Villa et al. 2019). Brackish food resources (mangroves, crustaceans, ctenophores) (Limpus and Limpus 2000; Arthur et al. 2008). Shelter for resting and predator avoidance (Chambault et al. 2020; Milly et al. 2002).
Geoscience Australia (Dyall et al. 2005)	Geomorphic habitats of Australia: ‘Geohabitat’	30 m	Static	2005	Coastal national	Overlap with polygon feature	Structural features for foraging, resting and predator avoidance (Chambault et al. 2020; Milly et al. 2002).
Marine monitoring program (TropWATER) (Gruber et al. 2024)	Long‐term frequency of water types 1&2: ‘Acute flood frequency’	250–1000 m (MODIS and Sentinel)	Static	2002–2022/23 summer	GBR	Extracted values at corresponding pixels (in space only)	Sediment and chemical influx and resuspension affect primary producers (Campbell and McKenzie 2004) and are associated with toxicity and pathogens (Flint et al. 2015, 2010; Villa et al. 2019). Acute events.
Marine monitoring program (TropWATER) (Gruber et al. 2024)	Long‐term exposure to above guideline value concentrations of land‐sourced pollutants: ‘Chronic floodwater exposure’	250–1000 m (MODIS and Sentinel)	Static	2002–2022/23 summer	GBR	Extracted values at corresponding pixels (in space only)	Sediment and chemical influx and resuspension affect primary producers (Campbell and McKenzie 2004) and are associated with toxicity and pathogens (Flint et al. 2015, 2010; Villa et al. 2019). Chronic conditions.
Beaman (2017)	Bathymetry, Rugosity, Slope	100 m	Static	2018	GBR	Extracted values at corresponding pixels (in space only). Ruggedness was calculated with QGIS ‘Terrain Ruggedness Index’ and Slope with QGIS Raster analysis ‘Slope’ tool.	Green turtles in foraging grounds throughout coastal Queensland and the GBR most frequently occupy depths 0‐30 m and rarely below 60 m (Limpus et al. 2013). Rugosity and slope may be indicative of geomorphic habitat type, physical structures and benthic communities.

We sampled values of each environmental predictor for each presence and pseudo‐absence point corresponding to their timestamp and location. For static datasets (a single, non‐time‐varying layer) (Table 1) we used terra::extract() for grid data, and sp.::over() for shapefile (categorical) data. We extracted values of eReefs variables via a custom R script to query the AIMS THREDDS server. We downloaded annual mangrove maps (Lymburner et al. 2020) from the Digital Earth Australia data cube using Dask and used these to generate annual maps of Euclidean distance to pixels of at least 20% canopy cover [i.e., minimum class where mangroves were present in the mangrove dataset (Lymburner et al. 2020)] in QGIS. Additionally, we generated 30 m (to match the high resolution of bathymetry data) grids of distance to recreational boating facilities, distance to coast, distance to reefs and distance to rivers in QGIS (details in Table 1). Prior to modelling, we evaluated the temporal coverage of the variables derived from the eReefs biogeochemical model with data hosted on the THREDDS server only being available from late 2010 until April 2019. We therefore removed presences and pseudo‐absences corresponding with timestamps after April 2019, corresponding to entire tracks of 22 turtles that were tracked in early 2010 or after April 2019. The final collection of turtle tracks used for model development is summarised in Appendix S1.

We evaluated environmental separation, the environmental conditions at presences compared with pseudo absences, using Bhattacharyya's coefficient, where 0 is entirely dissimilar and 1 is identical. We examined pairwise correlations of predictor variables and did not include any two predictors that were more than 70% correlated in subsequent modelling.

#### 2.4.3 Modelling Approach

We used boosted regression trees to develop the telemetry‐based habitat models for (1) modified (Port Curtis) and (2) unmodified (Shoalwater Bay and foraging sites of Raine Island turtles) sites separately, with the gbm.fixed() function of the dismo R package (Hijmans et al. 2023). These models use presences and pseudo‐absences as the response, and the environmental values as predictors. This approach assumes that presences and pseudo‐absences are not autocorrelated (Hazen et al. 2021; Elith et al. 2008). For each model formulation we used five‐fold cross validation, whereby the dataset was split into five parts, with each part acting as a test set for one model, and the remaining parts as the training set. We used a tree complexity of five, a learning rate of 0.005, a maximum of 10,000 trees and a bag fraction of 0.75. We plotted the relationship of each predictor in the input data with the response to inform our specification of the var.monotone argument of gbm.fixed(). The initial folds were generated including all of the input predictors and a large (> 10,000) number of trees. We then re‐ran the cross‐validation, removing those predictors that were relatively unimportant (in the lowest 10% in every fold) to simplify the model and pruned using the out of bag method to adjust the n.trees parameter to encompass the best number of iterations of all the folds. Variable importance is assessed as the total contribution by each variable to a reduction in the loss function.

We evaluated model performance with averages of the performance metrics from the five folds: predictive skill with area under the curve (AUC), true skill statistic (TSS), true positive rate (TPR) and explanatory power with percent deviance explained. The final model was trained on 100% of the input data with the refined hyper‐parameters obtained during the cross‐validation process.

To investigate environmental predictors in relation to foraging habitat for green turtles, we obtained a ranking of the most influential environmental predictors. We examined the shape of the relationship with the response for each predictor with variable importance greater than 100/number of predictors in the model, with partial deviance plots. Influential two‐way interactions were identified with gbm.interactions().

To estimate the distribution of suitable foraging habitat for green turtles in the Great Barrier Reef inshore region, we first created grids of each environmental predictor, with matching projection (EPSG: 3577), origin, resolution (500 m pixels) and extent (clipped to the extent of the Carter et al. 2021 seagrass dataset).

To evaluate how the extent and distribution of suitable foraging habitat in the unmodified areas of the region changed over time, we compared the area and spatial distribution of predictions of probability of foraging turtle presence for two time periods, December 2010 and December 2022, using the unmodified model. We deemed this model the most appropriate to generalise habitat conditions for all turtles across the modelled range. We evaluated these differences by varying influential time‐varying (not static) predictors underpinning the model. Variables from the eReefs biogeochemical model in 2022 were available at daily, rather than monthly, aggregations.

Finally, to quantify the area of suitable habitats within or outside of protected areas, we calculated the proportion by area of the predicted suitable foraging habitat, that is, pixels with a probability of foraging turtle presence > 0.5 according to the unmodified model, that was located within the Great Barrier Reef Marine Park boundary, specific marine park zones and designated port areas. The scripted R workflow for this study is available in a public Github repository as per Appendix S4.

## 3 Results

Turtle presences and pseudo‐absences were concentrated less than 50 km from the coast (all 172,111 presences and 1,513,266 of 1,513,281 pseudoabsences), largely in shallow bays and inlets (Appendix S2). Presences of some individuals fell upstream of river mouths (*n* = 15 turtles) and on coral reefs (*n* = 12 turtles). The sampling of presences and pseudo‐absences in this study was skewed to turtles foraging in Port Curtis (*n* = 68 turtles with adequate state‐space models, see methods) over the other capture sites. The spread of pseudo‐absences resembled the distribution of the presences, though less concentrated. Environmental separation is summarised in Table 2.

**TABLE 2:** Bhattacharyya's coefficient (0–1, where 0 is entirely dissimilar and 1 is identical) for the four most influential variables identified in each habitat model, indicating environmental separation between presences and pseudo‐absences.

Variable rank importance	Correlated random walks	
	Modified	Unmodified
1	Distance to boat ramps 0.77	Distance to reefs 0.97
2	Temperature 0.99	Mean saltwater velocity 0.70
3	Distance to reefs 0.97	Distance to boat ramps 0.77
4	Salinity 0.99	Acute flood frequency 0.15

**FIGURE 2:**
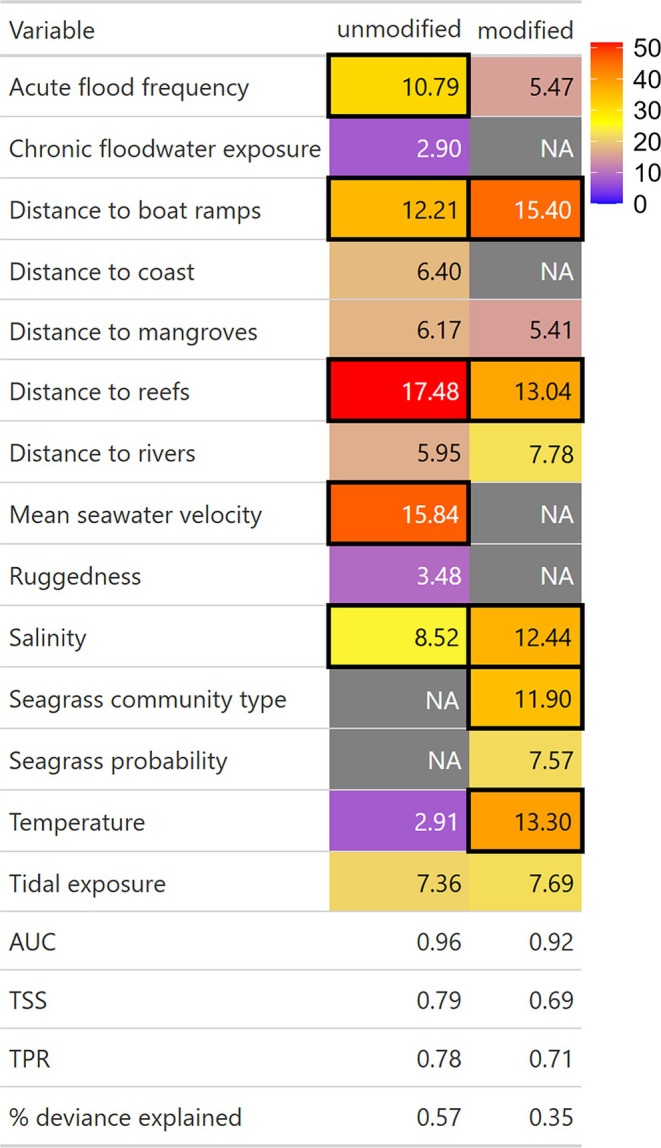
Relative importance of environmental predictor variables in telemetry‐based habitat models using pseudo‐absences from correlated random walks at modified (Port Curtis) or unmodified (Shoalwater Bay and foraging sites post‐nesting from Raine Island) sites. Colour is indicative of relative importance. Variables with greater than null explanatory power (relative importance > 100/number of variables in the model) are outlined in black. Evaluation metrics of each model are provided (% deviance explained = (null deviance—residual deviance)/null deviance; AUC, area under the curve; TPR, true positive rate; TSS, true skill statistic).

The unmodified and modified models both had an AUC > 0.90. Predictive skill was TSS = 0.79 for the unmodified and TSS = 0.69 for the modified model. Deviance explained was > 0.57 for the unmodified model and 0.35 for the modified model (Figure 2).

### 3.1 What Characteristics Make Foraging Habitat Suitable for Green Turtles?

Distance to boat ramps, temperature, distance to reefs, salinity and seagrass community type were influential variables in modified habitats. In unmodified habitats, distance to reefs, mean seawater velocity, distance to boat ramps, acute flood frequency and salinity were the influential variables. The predictor values appended to presences and pseudo‐absences that were all at least 70% correlated with each other were distance to coast, distance to mangroves and acute flood frequency. Distance to rivers was correlated with distance to boat ramps. Ruggedness and slope were also correlated.

The shape of the relationships between influential variables and the probability of turtle presence for each model is depicted in Appendix S5. Influential variables that also appeared among the five highest ranking two‐way interactions for that model are listed in Appendix S6. The seagrass communities (Carter et al. 2021) predictive of turtle presence from each model are summarised in Appendix S7.

### 3.2 Where Is Suitable Foraging Habitat for Green Turtles in the Great Barrier Reef Inshore Region?

The unmodified model is the most appropriate for generalising the environmental characteristics underpinning habitat suitability across the modelled extent prior to human modification, and has pseudo‐absences that represent environmental separation at the scale at which turtles are making movement decisions in foraging grounds. This model identified known reefs and sheltered coastal inlets along the entire latitudinal range of the modelled region as being suitable foraging habitat for green turtles in 2022. It excluded deep areas between the coast and mid and offshore reefs including the Capricorn Bunker group of islands, which are identified as important foraging areas for the southern Great Barrier Reef stock by the Queensland Marine Turtle Conservation Strategy (Queensland Department of Environment and Science 2021).

The unmodified model predicted 1.6% of the modelled area as being suitable habitat for foraging green turtles in 2022 (3621 km^2^ had more than 50% predicted probability of turtle presence). In comparison, the modified model predicted 0.2% of the modelled area as being suitable.

### 3.3 Has the Extent and Distribution of Suitable Foraging Habitat in the Region Changed Over Time?

**FIGURE 3:**
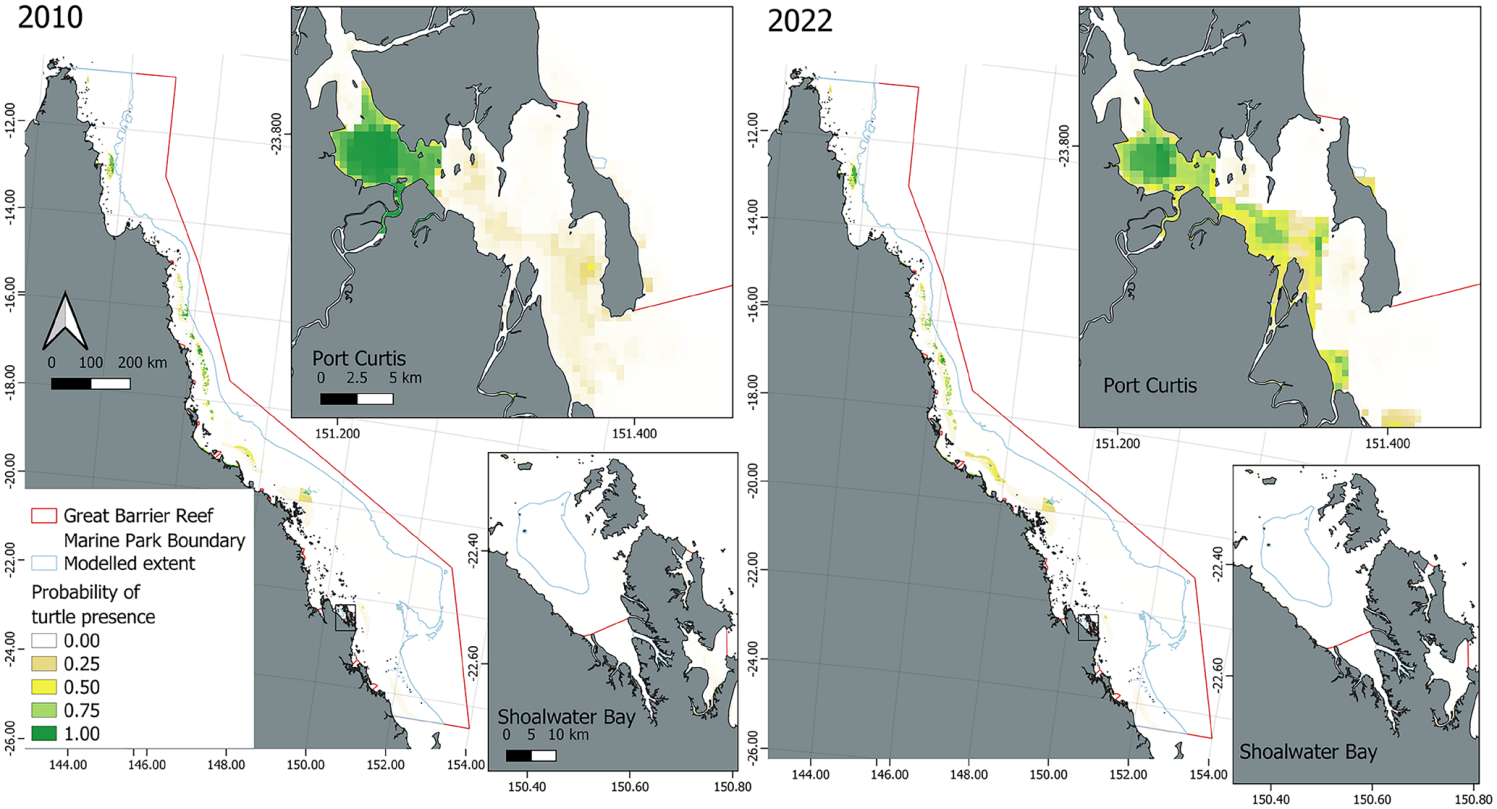
Distribution of suitable foraging habitat for green turtles in December 2022 and December 2010 indicating designated port areas and marine park boundary, predicted from telemetry‐based habitat models with pseudoabsences generated from correlated random walks in unmodified habitats.

**FIGURE 4:**
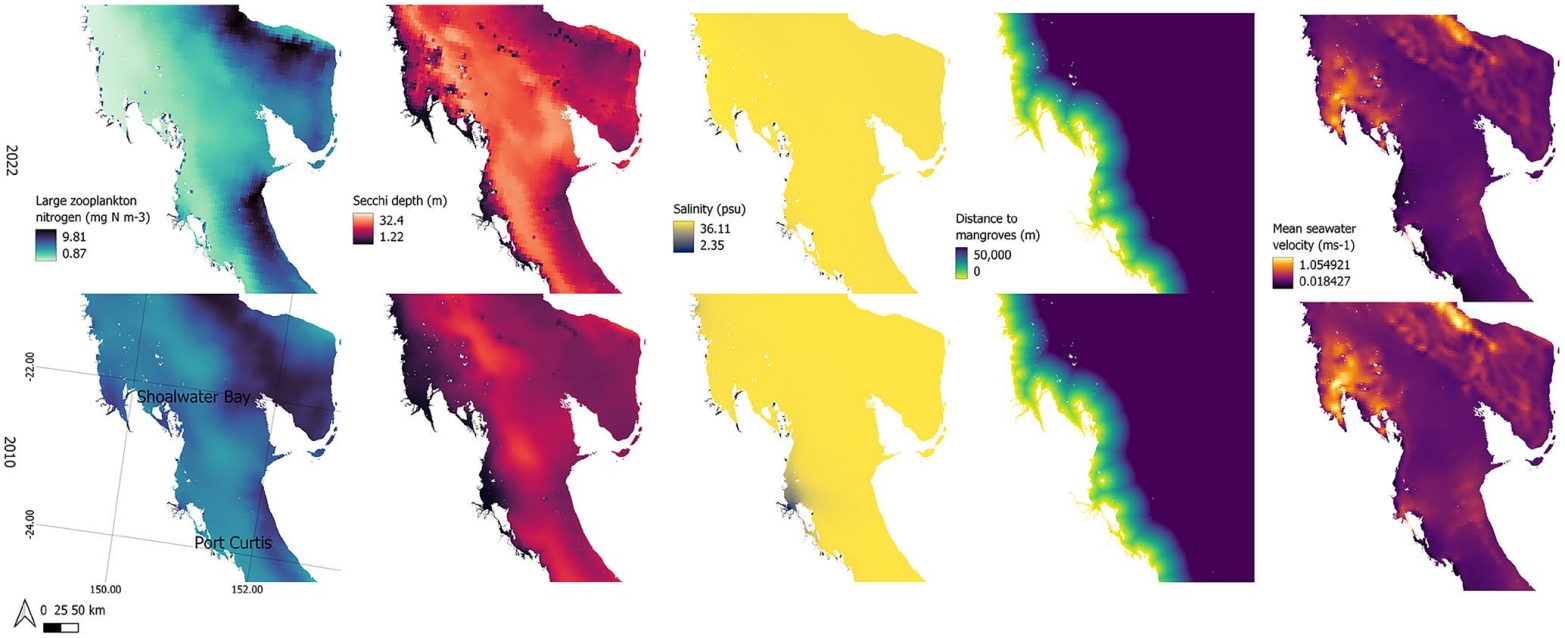
Time‐varying covariates large zooplankton nitrogen, Secchi depth, salinity, distance to mangroves and mean seawater velocity in December 2010 and December 2022.

Our unmodified model suggested that the total area where predicted probability of turtle presence was more likely than not (i.e., probability of presence > 0.5), increased by around 26.7%, from 2952.5 km^2^ in 2010 to 3621 km^2^ in 2022 (Figure 3). The model predicted that 100% of the predicted suitable habitat area was within 50 km of the coast in 2010 and 2022. In 2010, suitable habitat was distributed less broadly both near mid‐shelf reefs, and closer to the coast than in 2022 (Figure 3). The time‐varying influential predictors in this model (having variable importance score greater than 100/number of predictors) were mean seawater velocity and salinity. Temporal differences could also be the result of the cumulative effect of several lower‐ranked time‐varying covariates‐ namely temperature and distance to mangroves (Figure 4).

### 3.4 How Much of Suitable Foraging Habitat Is Protected?

Potential benefits of zoning to green turtles include protection of benthic habitat from trawling, reduced risk of boat strike, reduced risk of bycatch and entanglement in fishing gear, reduced risk of traditional harvest and reduced alterations to trophic webs from extractive activities. Of the total suitable habitat in the modelled area, 46.6% fell within Habitat Protection Zones (trawling not permitted), 16.5% in marine National Park (along with Presearvation and Scientific Research Sones, these are no‐take), 14.1% in Conservation Park (limited fishing activities), 20.8% in General Use Zones, 0.8% in Preservation Zones and the remainder in Buffer Zones based on the prediction from the 2022 unmodified model. Of total suitable foraging habitat, 5.2% fell within designated port areas. Noting that our modelled extent was clipped to match the extent of the input environmental data (Carter et al. 2021), 8.9% of identified suitable habitat fell inland of the Great Barrier Reef Marine Park boundary (i.e., within port areas and areas landward of the low water mark including rivers and estuaries).

## 4 Discussion

We created a dynamic regional distribution model of suitable inshore foraging habitats for green turtles in eastern Queensland, Australia. This fills a substantial gap in the existing knowledge for the species, where foraging distribution was previously defined by the locations of index sites, post‐nesting tracks of breeding females and opportunistic capture close to human populations (Queensland Department of Environment Science and Innovation, Convention on the Conservation of Migratory Species of Wild Animals (CMS) 2021). We expected green turtles to be distributed all along the coast of Queensland, with the highest density in mid reefs and lower density in the outer reefs. The predicted habitat distribution sensibly captured known shallow coastal areas used by foraging green turtles within the modelled region, including Port Curtis, Shoalwater Bay, Cleveland Bay, river mouths (Queensland Department of Environment and Science 2021; Limpus 2008) while excluding improbable areas (deep areas between the coast and the mid‐shelf reefs). The model did not capture known foraging locations greater than 50 km from the mainland coast. We ascertained that the total area of potentially suitable habitat has increased by 26.7% between 2010 and 2022, and the distribution of suitable habitats spread along the mid‐shelf. Given the established relationship between weather events and habitat quality for marine turtles in coastal foraging grounds (Limpus and Nicholls 2000; Cleguer et al. 2022; Shimada et al. 2016; Rasheed et al. 2014; Campbell and McKenzie 2004) we would expect changes in foraging habitat to align with weather events in confined coastal areas. However, away from coasts and across broad areas, particularly reefs, habitat change will likely occur with the speed of climate change.

We identified important variables that characterise suitable foraging habitats for green turtles. In general, among these variables, we identified the distance to reefs and variables correlated to distance to coastline, distribution of food resources (i.e., seagrass communities) and physical characteristics of those habitats (e.g., currents, salinity and temperature) as having the strongest influence on the probability of turtle presence. Surprisingly, our unmodified model did not include seagrass probability or community type as a major predictor, as would be expected from green turtle diets. In Queensland, green turtles consume a variety of seagrasses (*Halophila ovalis, Halodule uninervis, Zostera capricorni, Halophila spinulosa, Halodule pinifolia, Cymodocea serrulate, Thalassia temprichii, Enhalus acoroides*) and a range of red, brown and green algae (Bjorndal 1997; Forbes 1996; Brand‐Gardner et al. 1999; André et al. 2005; Arthur et al. 2009; Limpus et al. 1994; Whiting and Miller 1998). At some sites, green turtles consume mangrove roots, fruits and cotelydons (*Rhizophera* and *Avicenia* sp.), though these usually contribute to relatively small proportions of the diet (Limpus and Limpus 2000; Amorocho and Reina 2008, 2007). Macroplankton (*ctenophora*, fish and crustaceans) are occasionally consumed but can contribute substantially to an individual diet sample (Arthur et al. 2009). Diet items may be supported by specific physical characteristics, but those physical characteristics may not dictate turtle habitat distribution directly. For example, seagrass variables were not influential in the unmodified model. Seagrasses may dominate diets at seagrass meadows, and macroalgae at algal pastures and rocky reefs such as Heron/Wistari Reefs (Forbes 1994), and parts of Port Curtis (Prior et al. 2016) and Moreton Bay (Arthur et al. 2008). Because data were not available at a suitable scale and resolution, algal distribution was not included in our model, despite being likely to dominate diets of foraging turtles occupying reef habitats. Though the seagrass (Carter et al. 2021) dataset leverages the benthic light variable from eReefs, the Secchi depth did not appear influential in any model.

The influential physical characteristics we identified align with previously described requirements for the region; Green turtles in foraging grounds throughout coastal Queensland and the Great Barrier Reef most frequently occupy areas close to reefs and coastlines with depths 0‐30 m and rarely below 60 m, reflecting the spatial distribution of predicted habitat presented here (Limpus et al. 2013). Distance to boat ramps and distance to the coast were highly correlated to each other but rarely both appeared as highly influential predictors in a single model. This may be an artefact of the decision tree approach whereby if a split related to one of correlated variables explains a large amount of deviance, subsequent splits related to the other variable will have low explanatory power. Regardless, turtle presence was predicted by both nearshore and mid‐shelf habitats in our chosen model. Indices of flooding and poor water quality (i.e., flood plume frequency and exposure, acute flood frequency and Secchi depth) were not influential to our models. However, in Queensland, the leading cause of green turtle stranding in coastal populations is disease, presumably associated with chemical and sediment runoff from river outflows and seasonal fluctuations in temperature and rainfall (Villa et al. 2019; Flint et al. 2017). Artificial structures (excluding boat ramps) did not come out as influential variables in our model selection procedure, though these can provide a foraging habitat for green turtles by supporting epifauna, infauna and macroalgal growth and or providing structures for resting (Crear et al. 2017; Holloway‐Adkins and Dennis Hanisak 2017). The degree to which turtles use man‐made structures is likely linked to their risk of anthropogenic interaction, including boat strike and incidental capture, and would be a useful avenue for future projects.

The higher relative importance of seagrasses in modified compared to unmodified habitats according to the models may reflect the availability of alternative foraging resources to seagrasses, including algae, at sites other than Port Curtis, particularly at coral and rocky reefs. It may also reflect the low explanatory power of the global and modified models, for example, though at some sites green turtles consume substantially larger proportions of seagrass in their adult diet compared to during immature stages (Esteban et al. 2020; Arthur et al. 2008), the turtles tracked at unmodified sites included fewer juveniles than in modified sites.

Our modelling approach could be further improved by incorporating dynamic rather than static environmental data as it becomes available (e.g., built infrastructure has changed throughout the study period). We have included predictors with non‐causal relationships to our response and potentially redundant predictors (De Kort et al. 2020). We note that we were only able to make predictions of suitable habitat where and when there was sufficient environmental and tracking data. In marine environments, more data exist for areas close to coasts and in shallow water (Robinson et al. 2017) for example, the modelled (Carter et al. 2021) seagrass data was lacking for estuaries and beyond the mid‐shelf. Matching scale, resolution and extent across the environmental predictor datasets highlighted the mismatch between the fine spatial resolution achievable with tracking technology and the achievable resolution of spatial environmental data at large scales. This will likely be better matched in future as spatial data collection and modelling methods improve. For model improvement we suggest inclusion of variables like waves, sediment, predator distributions and connectivity. Our turtle locations were biassed towards tracked animals in Port Curtis, which was our only modified site. Therefore the modified model may reflect unique site characteristics at Port Curtis and is not appropriate for generalising habitat suitability to other modified sites. Our unmodified model was informed by tracks of nine individuals. The addition of tracking data from other unmodified foraging sites, or capture data from foraging site monitoring programmes, would likely improve reliability of our output; however, this data were not available for inclusion in the present study. This work is intended as a baseline upon which it would be possible to overlay anthropogenic threats layers to quantitatively assess susceptibility of turtle foraging habitat to these threats, similar to (Ferreira et al. 2021; Hazen et al. 2018; Maxwell et al. 2013). It provides a prediction in space and time of likelihood of occurrence of the species during foraging; it does not represent turtle density. Future attempts to quantify density could incorporate detectability measures or abundance estimates from in‐water capture into our approach (Guillera‐Arroita et al. 2015; Catry et al. 2023).

Green turtle foraging habitats were explicitly considered in the design of the Great Barrier Reef Marine Park zoning in 2004 (Dobbs et al. 2007). Though substantal protections are offered to turtles by the Great Barrier Reef Marine Park zones, with 17.3% of potential suitable habitat for green turtles in 2022 within no‐take areas, 46.6% was within Habitat Protection Zones, which until 2027 allow large mesh gill netting. The Australian and Queensland governments have commited to phasing out gill netting in the Great Barrier Reef World Heritage area by 2027 (Department of Agriculture and Fishing 2024). Only 14.1% of predicted habitat was within Conservation Park Zones, which allow limited fishing and collecting. 8.9% of predicted suitable habitat fell outside of the Marine Park boundary (i.e., in designated port areas or inland of mean high water). Our results suggest that the area and distribution of suitable habitat has changed from 2010 to 2022. Despite being within the Marine Park boundary, turtle habitats are susceptible to indirect effects of climate change and development, which can be exacerbated by human activities (e.g., runoff), as well as direct impact human‐wildlife interactions (e.g., boat strike). In designated port areas and upstream of rivers, anthropogenic use can be intensive, and there are competing needs for space between human uses and marine habitats. Thus, there is a need to evaluate the threat landscape in these areas. The predicted range of suitable foraging habitat we present does not equal the realised foraging range for this species, because anthropogenic activities are not encompassed in the input data. Designation of protected areas may not be appropriate or feasible due to conflicting environmental, economic, social and cultural interests in these areas. Therefore appropriate, effective and enduring conservation initiatives must consider and balance environmental objectives with information on human‐use.

Green turtles may reside in a single foraging area between breeding migrations (Limpus et al. 1994) and across decades (Shimada et al. 2019; Limpus et al. 2009). For green turtles, home ranges in foraging grounds are closely linked to foraging resource distribution (Hart et al. 2017; Stokes et al. 2019). The quality of available forage affects growth rates, age to maturity and reproductive output of the species (Chaloupka et al. 2004). Fidelity to foraging and nesting habitat may limit sea turtles' capacity to displace from depleted sites or recolonise recovered sites (Watanabe et al. 2011). Protection of a few targeted key sites is therefore likely to confer considerable conservation benefit (Schofield et al. 2013; Hays et al. 2014; Scott et al. 2012). However, our model predicted potentially suitable habitat for foraging green turtles, not all of which is likely to represent key high density foraging sites. Marine Protected Areas might not be appropriate over such vast areas or in low‐density foraging grounds for green turtles, and concentrating conservation efforts on small discrete areas may exacerbate the risk of unexpected impacts. Our results suggest that some foraging areas are more affected by changes over time in weather and temperature than others. While some major foraging habitats are affected, others which are less impacted might be more suited to protection over broader timescales. In addition, preventative interventions can help to target general habitat health rather than conservation of specific, high‐density areas for example, targeting sources of land‐based runoff through land restoration programmes to manage the impact of rainfall events on nearshore benthic habitats.

Considering this, dynamic management strategies may be appropriate in large areas where sea turtle aggregations are sparse. Dynamic management may involve implementation of temporally or spatially variable restrictions on take, recreational use (e.g., Go‐Slow Zones) or urban and industrial development in order to mitigate direct anthropogenic impacts to sea turtles and their habitats. Dynamic management with regular re‐evaluation is also pertinent considering long‐term changes to coastal and marine environments. However, realistic timeframes for ‘flexible’ interventions or protections are yet to be established for example, yearly management changes would be very challenging to implement. Despite local and regional conservation successes, mitigating impacts of habitat loss and deterioration over time on marine turtles is an ongoing global challenge (Hays and Hawkes 2018).

## Author Contributions

**Emily Webster:** conceptualization (lead), data curation (lead), formal analysis (lead), methodology (lead), visualization (lead), writing – original draft (lead), writing – review and editing (lead). **Stephanie Duce:** conceptualization (supporting), supervision (equal), visualization (supporting), writing – review and editing (supporting). **Colin Limpus:** conceptualization (supporting), funding acquisition (equal), project administration (equal), supervision (supporting), writing – review and editing (supporting). **Nicholas Murray:** conceptualization (supporting), methodology (supporting), supervision (supporting), writing – review and editing (supporting). **Toby Patterson:** conceptualization (supporting), funding acquisition (equal), project administration (equal), writing – review and editing (supporting). **Richard Pillans:** conceptualization (supporting), data curation (supporting), funding acquisition (supporting), methodology (supporting), project administration (supporting), writing – review and editing (supporting). **Takahiro Shimada:** data curation (supporting), funding acquisition (supporting), project administration (supporting), supervision (supporting). **Mark Hamann:** conceptualization (supporting), data curation (supporting), funding acquisition (equal), methodology (supporting), project administration (equal), supervision (lead), writing – review and editing (supporting).

## Funding

This work was supported by Shell's QGC Business, Australia Pacific LNG and Santos GLNG. GHD Group. The Centre for Tropical Water and Aquatic Ecosystem Research (TropWATER). Queensland Department of Environment, Tourism, Science and Innovation. Orica Limited. James Cook University. Gladstone Ports Corporation. CSIRO. GISERA Marine Project.

## Conflicts of Interest

The authors declare no conflicts of interest.

## Supporting information


**Appendices S1–S7:** ece373146‐sup‐0001‐AppendicesS1‐S7.docx.

## Data Availability

The data that support the findings of this study are available on Dryad at https://doi.org/10.5061/dryad.mcvdnck80. Code supporting analyses in the present study is available on Github at https://github.com/egwebster/SSM‐SDM‐public.
