# Supplementary material for: A Dynamic Foraging Habitat Distribution Estimate for Green Turtles in the Great Barrier Reef
Source: Ecol Evol. 2026 Feb 25;16(3):e73146. doi: 10.1002/ece3.73146 (PMC12936393; doi:10.1002/ece3.73146)
Supplement: Supplementary file 1 — Appendices S1–S7: ece373146‐sup‐0001‐AppendicesS1‐S7.docx. [file ECE3-16-e73146-s001.docx]

## Appendices

Appendix S1 Inventory of tracked green turtles with identifiers Primary tag (flipper tag), platform terminal transmitter (PTT) and tracker type. Maturity is coded as Adult (A), Subadult (SA) and Juvenile (J), and sex as male (M), female (F) or not determined (I)

| **Primary tag** | **PTT** | **Deploy site** | **Tag Type** | **Sex** | **Maturity** | **CCL (cm)** | **Year** | **Track Segments (n)** | **Duration (days)** | **Presences** | **Pseudo-absences** |
| --- | --- | --- | --- | --- | --- | --- | --- | --- | --- | --- | --- |
| K283 | 143704 | GL | SPLASH-10-F-296A | F | A | 98.1 | 2014 | 1 | 100 | 184 | 19088 |
| K28651 | 149082 | GL | SPLASH10-F-297A | M | A | 98.9 | 2015 | 2 | 174 | 318 | 32788 |
| K70229 | 133767 | GL | SPLASH-10-F-296A | F | A | 105.7 | 2013 | 3 | 395 | 184 | 19475 |
| K93086 | 96781 | GL | Sirtrack Fastloc | M | A | 85.6 | 2010 | 1 | 173 | 35 | 3303 |
| K93087 | 96777 | GL | Sirtrack Fastloc | M | A | 104.3 | 2010 | 5 | 240 | 69 | 7236 |
| QA13938 | 134182 | GL | SPLASH-10-F-296A | M | A | 95.4 | 2014 | 2 | 194 | 363 | 36368 |
| QA15576 | 45786 | RI | SPLASH10-F-334C | F | A | 102.2 | 2017 | 1 | 111 | 34 | 3385 |
| QA15580 | 45787 | RI | SPLASH10-F-334C | F | A | 106.2 | 2017 | 2 | 55 | 95 | 9403 |
| QA32523 | 133762 | GL | SPLASH10-F-297A | I | J | 49.1 | 2013 | 1 | 45 | 80 | 6606 |
| QA33327 | 133765 | GL | SPLASH-10-F-296A | M | A | 96.5 | 2013 | 1 | 66 | 130 | 12797 |
| QA33335 | 134180 | GL | SPLASH-10-F-296A | F | A | 89 | 2014 | 2 | 77 | 59 | 5750 |
| QA33342 | 133764 | GL | SPLASH-10-F-296A | F | A | 111 | 2013 | 1 | 105 | 209 | 20281 |
| QA33348 | 133769 | GL | SPLASH-10-F-296A | F | A | 107.3 | 2013 | 1 | 55 | 110 | 10817 |
| QA33349 | 133759 | GL | SPLASH10-F-297A | I | J | 42.6 | 2013 | 1 | 144 | 273 | 28196 |
| QA33350 | 133763 | GL | SPLASH10-F-297A | I | J | 42.1 | 2013 | 1 | 44 | 41 | 4348 |
| QA33368 | 133760 | GL | SPLASH10-F-297A | I | J | 46 | 2013 | 1 | 65 | 113 | 12728 |
| QA33394 | 133758 | GL | SPLASH10-F-297A | I | J | 43.6 | 2013 | 1 | 92 | 58 | 6946 |
| QA34529 | 133761 | GL | SPLASH10-F-297A | I | J | 47.9 | 2013 | 1 | 57 | 106 | 11244 |
| QA36851 | 96776 | SB | Sirtrack | M | A | 91.8 | 2014 | 3 | 87 | 142 | 13588 |
| QA36853 | 48862 | SB | Sirtrack | F | J | 95.2 | 2014 | 1 | 340 | 118 | 26161 |
| QA36875 | 134178 | GL | SPLASH-10-F-296A | M | A | 97.6 | 2014 | 1 | 161 | 288 | 28831 |
| QA43023 | 133770 | GL | SPLASH-10-F-296A | F | A | 102.7 | 2013 | 1 | 67 | 136 | 13313 |
| QA43063 | 133768 | GL | SPLASH-10-F-296A | M | A | 93.5 | 2013 | 1 | 69 | 136 | 13896 |
| QA43066 | 133766 | GL | SPLASH-10-F-296A | F | A | 105.7 | 2013 | 1 | 62 | 88 | 8642 |
| QA43123 | 149087 | GL | SPLASH10-F-297A | F | A | 108.2 | 2015 | 5 | 215 | 218 | 21736 |
| QA45524 | 134183 | GL | SPLASH-10-F-296A | F | A | 101.7 | 2014 | 3 | 166 | 199 | 20321 |
| QA45554 | 134184 | GL | SPLASH-10-F-296A | F | A | 116.6 | 2014 | 1 | 197 | 362 | 39060 |
| QA45566 | 134188 | GL | SPLASH-10-F-296A | F | A | 110.9 | 2014 | 3 | 90 | 128 | 12479 |
| QA45601 | 134185 | GL | SPLASH-10-F-296A | I | SA | 79 | 2014 | 3 | 182 | 223 | 22398 |
| QA45627 | 134181 | GL | SPLASH-10-F-296A | I | J | 63.1 | 2014 | 3 | 207 | 348 | 39616 |
| QA45654 | 134186 | GL | SPLASH-10-F-296A | I | J | 50.2 | 2014 | 1 | 256 | 66 | 6569 |
| QA45689 | 134187 | GL | SPLASH-10-F-296A | M | A | 102.5 | 2014 | 2 | 117 | 213 | 21945 |
| QA46109 | 96777 | SB | Sirtrack | M | A | 94.5 | 2014 | 1 | 472 | 14 | 1911 |
| QA46144 | 48861 | SB | Sirtrack | M | A | 90.2 | 2014 | 1 | 103 | 21 | 2100 |
| QA58206 | 149088 | GL | SPLASH10-F-297A | F | SA | 81.5 | 2015 | 3 | 135 | 203 | 21020 |
| QA58209 | 149081 | GL | SPLASH10-F-297A | M | A | 89.1 | 2015 | 1 | 94 | 186 | 18721 |
| QA58210 | 149086 | GL | SPLASH10-F-297A | M | SA | 80.1 | 2015 | 3 | 166 | 208 | 20308 |
| QA58211 | 149085 | GL | SPLASH10-F-297A | F | SA | 99.7 | 2015 | 1 | 129 | 216 | 21183 |
| QA58221 | 149080 | GL | SPLASH10-F-297A | F | A | 95.1 | 2015 | 4 | 171 | 242 | 24871 |
| QA58239 | 149083 | GL | SPLASH10-F-297A | M | SA | 77.8 | 2015 | 1 | 146 | 129 | 13746 |
| QA58284 | 149084 | GL | SPLASH10-F-297A | M | A | 92.2 | 2015 | 1 | 123 | 230 | 24202 |
| QA58291 | 149090 | GL | SPLASH10-F-297A | M | A | 94.1 | 2015 | 3 | 142 | 49 | 6405 |
| QA58295 | 149089 | GL | SPLASH10-F-297A | F | SA | 83.8 | 2015 | 1 | 156 | 209 | 20709 |
| QA64251 | 152716 | GL | SPLASH10-F-297A | F | SA | 75.7 | 2017 | 3 | 258 | 95 | 9416 |
| QA64291 | 152717 | GL | SPLASH10-F-297A | M | SA | 76.2 | 2017 | 1 | 142 | 9 | 3395 |
| QA64318 | 133758 | GL | SPLASH10-F-297A | F | A | 94.6 | 2017 | 1 | 71 | 137 | 14154 |
| QA64830 | 157925 | GL | SPLASH10-F-297A | M | A | 94.7 | 2016 | 3 | 122 | 22 | 9317 |
| QA64930 | 157926 | GL | SPLASH10-F-297A | F | A | 108.7 | 2016 | 4 | 139 | 152 | 16534 |
| QA64931 | 157927 | GL | SPLASH10-F-297A | F | A | 96.8 | 2016 | 1 | 84 | 81 | 8149 |
| QA64932 | 157929 | GL | SPLASH10-F-297A | F | A | 103.5 | 2016 | 1 | 73 | 118 | 11361 |
| QA64933 | 157928 | GL | SPLASH10-F-297A | M | SA | 85.3 | 2016 | 3 | 167 | 239 | 29140 |
| QA65085 | 157933 | GL | SPLASH10-F-297A | M | A | 96.4 | 2016 | 3 | 106 | 184 | 19507 |
| QA65088 | 157931 | GL | SPLASH10-F-297A | M | A | 101.7 | 2016 | 2 | 152 | 232 | 26238 |
| QA65177 | 157930 | GL | SPLASH10-F-297A | F | A | 114.6 | 2016 | 1 | 112 | 137 | 14908 |
| QA66466 | 157934 | GL | SPLASH10-F-297A | M | A | 92.9 | 2016 | 1 | 148 | 272 | 27911 |
| QA66526 | 157935 | GL | SPLASH10-F-297A | F | A | 97.3 | 2016 | 1 | 74 | 146 | 14837 |
| QA80426 | 45755 | RI | SPLASH10-F-334C | F | A | 98.1 | 2017 | 1 | 47 | 11 | 1091 |
| QA80430 | 45791 | RI | SPLASH10-F-334C | F | A | 102.6 | 2017 | 1 | 71 | 143 | 14292 |
| QA81295 | 45778 | RI | SPLASH10-F-334C | F | A | 102.7 | 2017 | 1 | 111 | 112 | 11076 |
| QA86025 | 40934 | GL | SPLASH10-BF-297B | M | SA | 96 | 2018 | 1 | 124 | 90 | 9592 |
| QA86189 | 61689 | GL | SPLASH10-BF-344E | M | A | 98 | 2018 | 1 | 73 | 148 | 14456 |
| QA86190 | 61690 | GL | SPLASH10-BF-344E | M | SA | 70.3 | 2018 | 2 | 175 | 280 | 27465 |
| QA86202 | 176006 | GL | SPLASH10-BF-297B | M | SA | 77.2 | 2018 | 1 | 188 | 307 | 36676 |
| QA86247 | 61692 | GL | SPLASH10-BF-344E | M | SA | 71.5 | 2018 | 1 | 111 | 213 | 20958 |
| QA86302 | 61691 | GL | SPLASH10-BF-344E | M | A | 91.3 | 2018 | 1 | 113 | 63 | 13789 |
| QA87017 | 64747 | GL | SPLASH10-F-334D | I | SA | 67.2 | 2018 | 1 | 138 | 240 | 25553 |
| QA87018 | 64748 | GL | SPLASH10-F-334D | I | SA | 67.4 | 2018 | 1 | 170 | 157 | 15550 |
| T88971 | 157932 | GL | SPLASH10-F-297A | F | A | 98.6 | 2016 | 1 | 156 | 301 | 31165 |
|  | 126272 | GL | SPLASH10-F | F | A | 106.2 | 2013 | 2 | 203 | 267 | 25080 |
|  | 126273 | GL | SPLASH10-F | F | SA | 101.6 | 2013 | 5 | 278 | 40 | 8775 |
|  | 126274 | GL | SPLASH10-F | F | A | 113.8 | 2013 | 2 | 165 | 178 | 27847 |
|  | 126275 | GL | SPLASH10-F | M | J | 58.8 | 2013 | 3 | 383 | 228 | 25720 |
|  | 126276 | GL | SPLASH10-F | M | J | 54.6 | 2013 | 3 | 160 | 75 | 7426 |
|  | 131862 | GL | SPLASH10-F | I | J | 52.1 | 2013 | 1 | 114 | 81 | 12162 |
|  | 131868 | GL | SPLASH10-F | M | A | 97.7 | 2013 | 3 | 243 | 376 | 39060 |
|  | 131869 | GL | SPLASH10-F | F | A | 96.2 | 2013 | 2 | 78 | 114 | 11636 |
|  | 131872 | GL | SPLASH10-F | I | J | 52.7 | 2013 | 1 | 191 | 283 | 36831 |

Appendix S2 Locations of green turtles’ presences from movement persistence modelling and pseudo-absences from simulated correlated random walks, used as inputs in telemetry-based habitat models, calculation of pseudo-absences types is depicted conceptually in the right hand panels, adapted from (22)


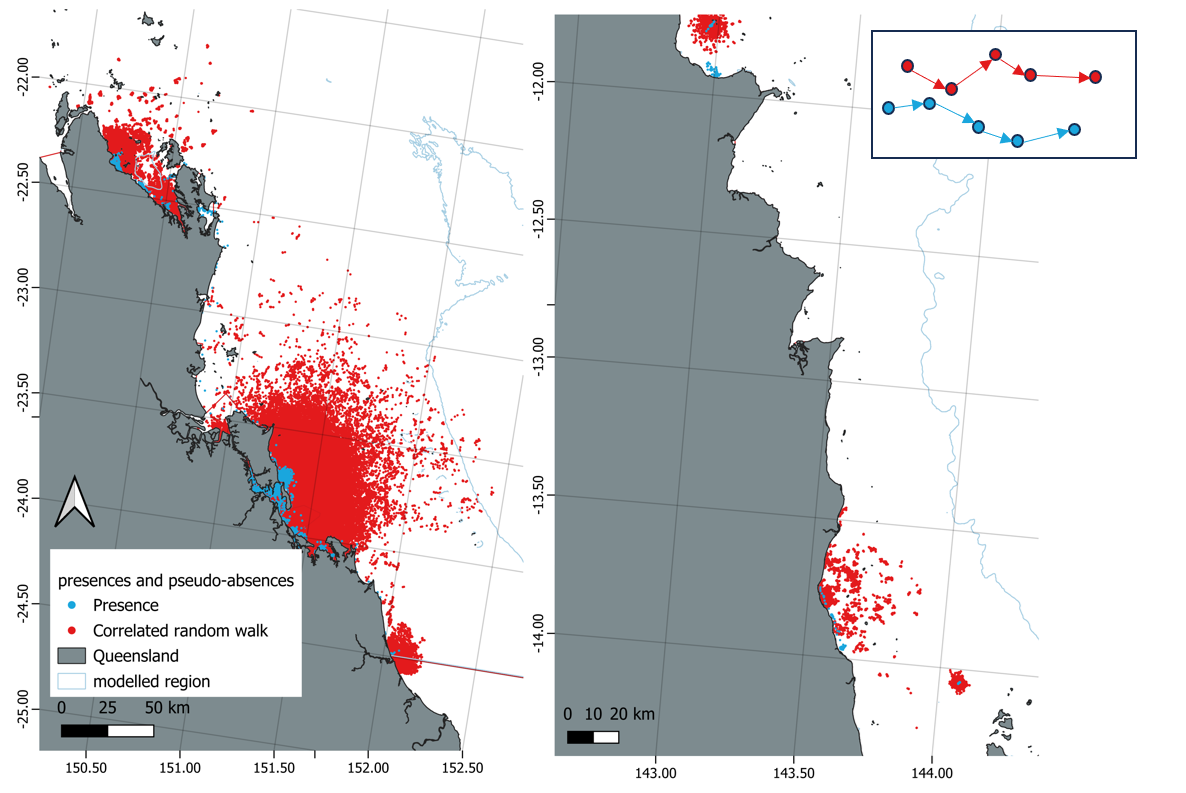


Appendix S3 Correlation matrix of environmental variables

|  | bathymetry | distance to rivers | distance to reefs | distance to coast | distance to boat ramps | seagrass probability | tidal exposure | acute flood frequency | chronic exposure to floodwater | ruggedness | slope | distance to mangroves | salinity | temperature | mean seawater velocity | mean windspeed | zooplankton nitrogen | EFI | Secchi |
| --- | --- | --- | --- | --- | --- | --- | --- | --- | --- | --- | --- | --- | --- | --- | --- | --- | --- | --- | --- |
| bathymetry | 1.00 | -0.42 | -0.33 | -0.75 | -0.30 | -0.03 | 0.35 | 0.80 | 0.76 | 0.08 | 0.08 | -0.75 | 0.01 | 0.00 | -0.25 | -0.26 | -0.07 | 0.11 | -0.72 |
| distance to rivers | -0.42 | 1.00 | 0.08 | 0.49 | 0.85 | 0.21 | -0.13 | -0.60 | -0.57 | -0.17 | -0.16 | 0.63 | -0.22 | 0.20 | 0.19 | 0.11 | -0.09 | -0.08 | 0.44 |
| distance to reefs | -0.33 | 0.08 | 1.00 | 0.50 | 0.21 | 0.31 | 0.03 | -0.48 | -0.26 | -0.14 | -0.14 | 0.34 | -0.10 | 0.02 | 0.30 | -0.03 | 0.24 | 0.12 | 0.16 |
| distance to coast | -0.75 | 0.49 | 0.50 | 1.00 | 0.37 | 0.27 | -0.20 | -0.84 | -0.72 | -0.24 | -0.22 | 0.84 | -0.03 | -0.02 | 0.33 | 0.22 | 0.12 | -0.10 | 0.67 |
| distance to boat ramps | -0.30 | 0.85 | 0.21 | 0.37 | 1.00 | 0.14 | -0.04 | -0.46 | -0.45 | -0.17 | -0.15 | 0.49 | -0.21 | 0.22 | 0.36 | 0.01 | -0.06 | 0.00 | 0.26 |
| seagrass probability | -0.03 | 0.21 | 0.31 | 0.27 | 0.14 | 1.00 | 0.20 | -0.34 | -0.19 | -0.37 | -0.35 | 0.22 | -0.14 | 0.04 | 0.12 | 0.05 | 0.13 | 0.01 | 0.15 |
| tidal exposure | 0.35 | -0.13 | 0.03 | -0.20 | -0.04 | 0.20 | 1.00 | 0.15 | 0.22 | 0.00 | -0.01 | -0.18 | -0.04 | 0.02 | -0.05 | -0.08 | 0.03 | 0.06 | -0.21 |
| acute flood frequency | 0.80 | -0.60 | -0.48 | -0.84 | -0.46 | -0.34 | 0.15 | 1.00 | 0.79 | 0.22 | 0.21 | -0.84 | 0.10 | -0.05 | -0.30 | -0.24 | -0.09 | 0.10 | -0.74 |
| chronic exposure to floodwater | 0.76 | -0.57 | -0.26 | -0.72 | -0.45 | -0.19 | 0.22 | 0.79 | 1.00 | 0.30 | 0.28 | -0.72 | 0.08 | -0.03 | -0.35 | -0.27 | -0.05 | 0.11 | -0.75 |
| ruggedness | 0.08 | -0.17 | -0.14 | -0.24 | -0.17 | -0.37 | 0.00 | 0.22 | 0.30 | 1.00 | 0.97 | -0.19 | 0.04 | 0.01 | -0.21 | -0.09 | -0.05 | 0.03 | -0.20 |
| slope | 0.08 | -0.16 | -0.14 | -0.22 | -0.15 | -0.35 | -0.01 | 0.21 | 0.28 | 0.97 | 1.00 | -0.17 | 0.04 | 0.01 | -0.20 | -0.08 | -0.05 | 0.03 | -0.18 |
| distance to mangroves | -0.75 | 0.63 | 0.34 | 0.84 | 0.49 | 0.22 | -0.18 | -0.84 | -0.72 | -0.19 | -0.17 | 1.00 | -0.07 | 0.01 | 0.34 | 0.22 | 0.13 | -0.10 | 0.63 |
| salinity | 0.01 | -0.22 | -0.10 | -0.03 | -0.21 | -0.14 | -0.04 | 0.10 | 0.08 | 0.04 | 0.04 | -0.07 | 1.00 | 0.00 | 0.02 | 0.10 | -0.42 | -0.16 | 0.13 |
| temperature | 0.00 | 0.20 | 0.02 | -0.02 | 0.22 | 0.04 | 0.02 | -0.05 | -0.03 | 0.01 | 0.01 | 0.01 | 0.00 | 1.00 | -0.02 | 0.14 | -0.41 | 0.07 | 0.00 |
| mean seawater velocity | -0.25 | 0.19 | 0.30 | 0.33 | 0.36 | 0.12 | -0.05 | -0.30 | -0.35 | -0.21 | -0.20 | 0.34 | 0.02 | -0.02 | 1.00 | 0.13 | 0.16 | 0.14 | 0.15 |
| mean windspeed | -0.26 | 0.11 | -0.03 | 0.22 | 0.01 | 0.05 | -0.08 | -0.24 | -0.27 | -0.09 | -0.08 | 0.22 | 0.10 | 0.14 | 0.13 | 1.00 | -0.07 | 0.14 | 0.20 |
| zooplankton nitrogen | -0.07 | -0.09 | 0.24 | 0.12 | -0.06 | 0.13 | 0.03 | -0.09 | -0.05 | -0.05 | -0.05 | 0.13 | -0.42 | -0.41 | 0.16 | -0.07 | 1.00 | 0.09 | -0.14 |
| EFI | 0.11 | -0.08 | 0.12 | -0.10 | 0.00 | 0.01 | 0.06 | 0.10 | 0.11 | 0.03 | 0.03 | -0.10 | -0.16 | 0.07 | 0.14 | 0.14 | 0.09 | 1.00 | -0.29 |
| Secchi | -0.72 | 0.44 | 0.16 | 0.67 | 0.26 | 0.15 | -0.21 | -0.74 | -0.75 | -0.20 | -0.18 | 0.63 | 0.13 | 0.00 | 0.15 | 0.20 | -0.14 | -0.29 | 1.00 |

Appendix S4 Availability of scripted R workflow

| The scripted workflow for the analysis presented in this study is available at https://github.com/egwebster/SSM-SDM-public |
| --- |

Appendix S5 Partial plots depicting the relationship of environmental variables with greater than null explanatory power (relative importance > 100/number of variables in model) to probability of green turtle presence for habitat models generated with pseudo-absences with correlated random walks in A unmodified and B modified habitats. *yhat* is the log-odds of the probability of turtle presence being 1. NSG stands for “non-seagrass habitat”, (E=estuary, C=coastal, R=reef, I=intertidal, S=subtidal)


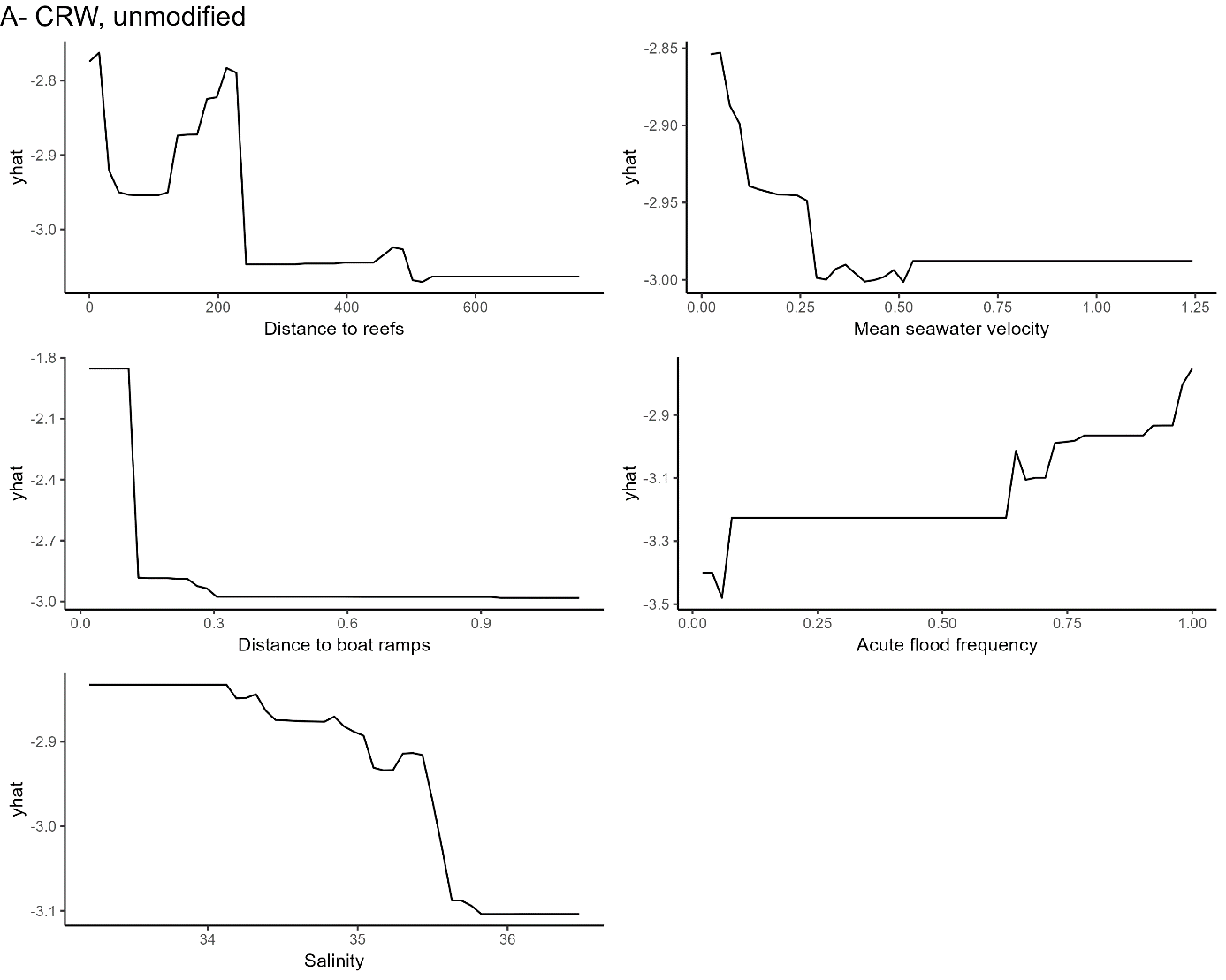


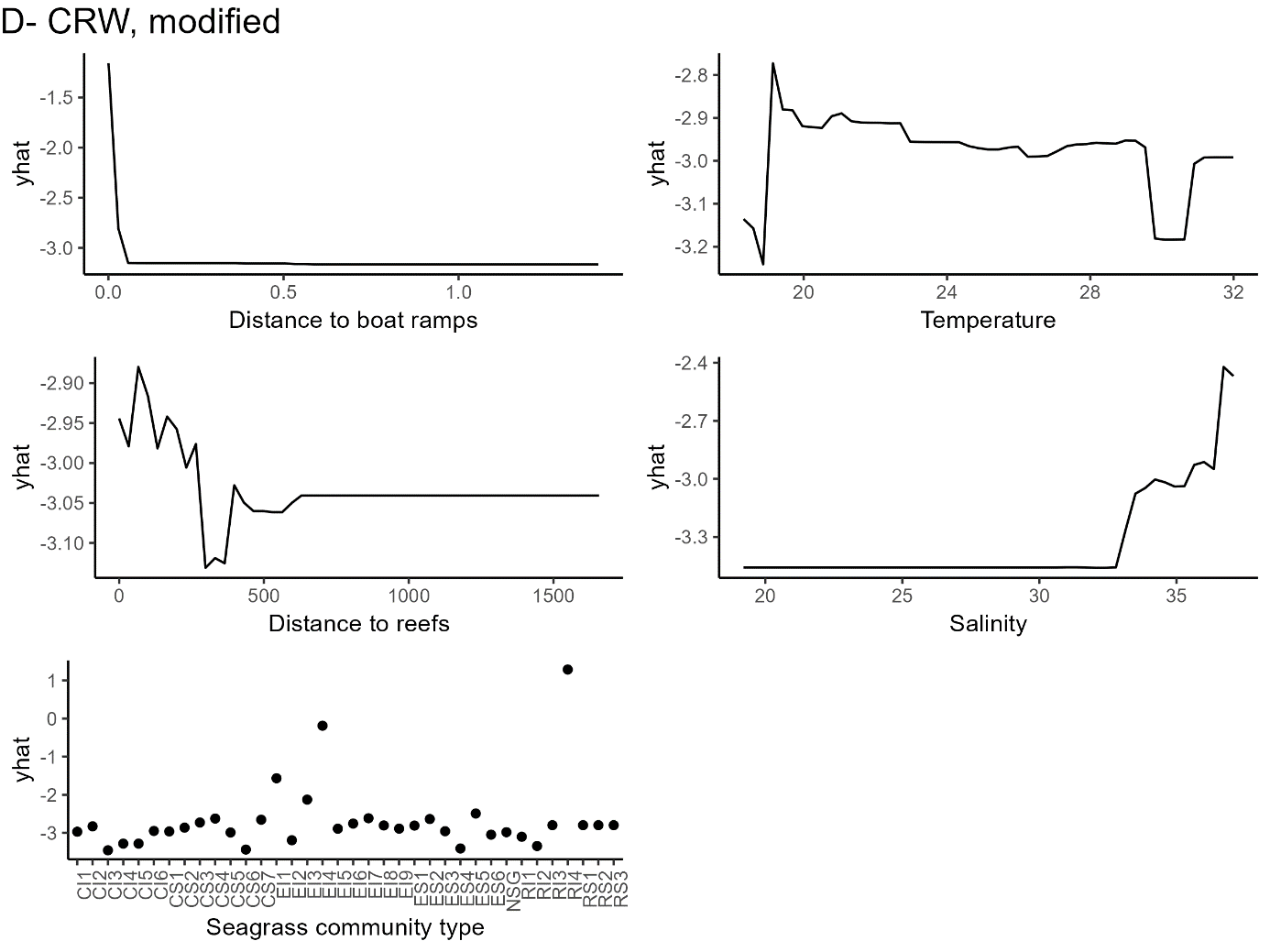


Appendix S6 Two-way interactions involving any influential predictors, ranked in top five

| modified | unmodified |
| --- | --- |
| Salinity X Temperature | Distance to reefs X Distance to rivers |
| Temperature X Distance to reefs | Distance to rivers X Acute flood frequency |
| Salinity X Distance to reefs | Acute flood frequency X Salinity |
| Seagrass community type X Distance to boat ramps | Mean seawater velocity X Distance to boat ramps |
| Seagrass community type X Tidal exposure | Distance to boat ramps X Distance to mangroves |

Appendix S7 Ranked list of top 10 seagrass community types (27) predictive of green turtle presence as identified in each habitat model (E=estuary, C=coastal, R=reef, I=intertidal, S=subtidal). Seagrass community type was not identified as an influential predictor in the unmodified model

| Rank of most predictive | modified | unmodified |
| --- | --- | --- |
| 1 | RI4 |  |
| 2 | EI4 |  |
| 3 | EI1 |  |
| 4 | ES3 |  |
| 5 | ES5 |  |
| 6 | CS4 |  |
| 7 | ES2 |  |
| 8 | CS7 |  |
| 9 | CS3 |  |
| 10 | EI6 |  |
